# Minds and Brains, Sleep and Psychiatry

**DOI:** 10.1176/appi.prcp.20200023

**Published:** 2020-11-10

**Authors:** J. Allan Hobson, Jarrod A. Gott, Karl J. Friston

**Affiliations:** ^1^ Division of Sleep Medicine Harvard Medical School Boston Massachusetts; ^2^ Donders Institute for Brain, Cognition and Behaviour Radboud University Nijmegen; ^3^ The Wellcome Centre for Human Neuroimaging University College London London

## Abstract

**Objective:**

This article offers a philosophical thesis for psychiatric disorders that rests upon some simple truths about the mind and brain. Specifically, it asks whether the *dual aspect monism*—that emerges from sleep research and theoretical neurobiology—can be applied to pathophysiology and psychopathology in psychiatry.

**Methods:**

Our starting point is that the mind and brain are emergent aspects of the same (neuronal) dynamics; namely, the brain–mind. Our endpoint is that synaptic dysconnection syndromes inherit the same dual aspect; namely, aberrant inference or belief updating on the one hand, and a failure of neuromodulatory synaptic gain control on the other. We start with some basic considerations from sleep research that integrate the phenomenology of dreaming with the neurophysiology of sleep.

**Results:**

We then leverage this treatment by treating the brain as an organ of inference. Our particular focus is on the role of precision (i.e., the representation of uncertainty) in belief updating and the accompanying synaptic mechanisms.

**Conclusions:**

Finally, we suggest a dual aspect approach—based upon belief updating (i.e., mind processes) and its neurophysiological implementation (i.e., brain processes)—has a wide explanatory compass for psychiatry and various movement disorders. This approach identifies the kind of pathophysiology that underwrites psychopathology—and points to certain psychotherapeutic and psychopharmacological targets, which may stand in mechanistic relation to each other.

## HIGHLIGHTS

- The ‘mind’ emerges from Bayesian belief updating in the ‘brain’
- Psychopathology can be read as aberrant belief updating.
- Aberrant belief updating follows from any neuromodulatory synaptopathy

The mind–brain problem is perpetuated by Cartesian dualism: we tend to think of mind and brain as quintessentially different kinds of things, correlated perhaps, but not two physical aspects of a unified system (1, 2). Working from the perspectives of sleep science and theoretical neurobiology, we have pursued the hypothesis that mind is a brain function (3). This essay attempts to unpack the implications of this bold but thoroughly justified move for medicine in general—and for neurology and psychiatry in particular. Since these considerations arise from the analysis of consciousness, we situate our arguments in that context and tie any clinical conclusions to it. In brief, we address the mind–brain problem from two complementary stances: namely, neurophysiology and theoretical neurobiology.

Our *theoretical foundation* derives from the work of Hermann von Helmholtz, a neurologically fluent physiologist who first suggested that the brain predicts the consequences of its sensorimotor activity (4, 5, 6). We elaborate the ensuing (free energy) principle in terms of Bayesian inference, which likens the brain–mind to a scientist seeking evidence for her hypotheses (7, 8). In short, our foundational assumption is that consciousness is itself a scientific enterprise (9).

*Our neurophysiological foundation* regards the brain as a physical system composed of billions of neurons, communicating via electrical and chemical signals—many of which have been rigorously characterized. Neuronal activity gives rise to subjective experience, which is conceived of as a kind of physics. The easiest way to appreciate the relationship of the brain to its mind is to consider the brain as the “material” that responds to biophysical forces and the “mind” as the belief updating driven by the same forces (10).

An early breakthrough in integrative brain–mind science was afforded by the discovery of rapid eye movement (REM) sleep and its concomitant hallucinoid dreaming (11, 12, 13, 14). Control of REM‐dreaming—by chemically specific neurons of the pontine brain stem—established the causal connection between basic neurophysiology and dream phenomenology (13, 15, 16, 17, 18, 19, 20, 21). Other conscious states, such as waking and non‐REM (NREM) sleep, derive from changes in the same neurophysiological factors. The details of these facts are debated but no‐one contests their importance to the control of conscious processes. We have reviewed the mathematical and neurophysiological foundations of dream phenomenology in a series of previous publications (3, 19, 22). Here, we revisit the conclusions from point of view of psychiatry. In what follows, we briefly review the philosophy and physiology of sleep, with a special focus on lucid dreaming and the activation, input and modulation (AIM) model. In what follows, some terms—like “beliefs”—are used in a technical sense that can depart from their folk psychology meaning. Key terms are listed in a glossary.

## Philosophy and Physiology

The radical enlightenment philosopher, Benedict Spinoza, first formulated dual aspect monism in his treatise on Ethics (23). Following Spinoza, we adopt dual aspect monism as follows: the brain–mind is a unified system with two aspects—an objective brain and a subjective mind. We further commit to the notion that there can be no mind in the absence of brain. This eliminates the dualistic assumption of non‐physical causation of mental phenomena. Since both aspects of the brain–mind system are physical, they can be mutually causal. This means that the scientific investigation of the mind is at once the investigation of the brain. From a philosophical perspective, one might argue that Cartesian dualism—as a de facto or legacy position—is still a serious obstacle to progress in consciousness science. Dual aspect monism and its manifold instantiations, some of which are illustrated here, offers a rejection of dualism, enabling a reconstruction of human self‐understanding.

There are many clinical implications of dual aspect monism: for example, (i) “mental illness” becomes a misnomer for psychiatric patients—a misnomer that denies any role for pathophysiology. (ii) A dissolution of the dichotomy between neurology and psychiatry, which divides two fields that belong together. (iii) The psychotic nature of REM‐dreaming; namely, an inherent propensity of the brain–mind for hallucinations and delusions (13, 14, 21, 24). In other words, REM‐dreaming may be viewed as a normal delirium, suggesting that the brain–mind evinces processes that are supposed to be exclusively pathological (25, 26). (iv) Revision of dream theory by viewing the unconscious brain–mind as a predictive model of the world, rather than an escape valve for unacceptable wishes (22). Finally, (v) the potentially potent role of psychotherapy, in resetting the disposition of the brain–mind.

### Neurophysiology

The neuronal doctrine of Ramon y Cajal (27) and the reflex doctrine of Charles Sherrington (28) dominated neuroscience from 1890 until about 1950. In the absence of sleep and dream science, Sigmund Freud was unable to complete his Project for a Scientific Psychology in 1895. Instead, he turned his attention to the psychanalytic interpretation of dreams (29), which he insisted was in no way neurological (30). The resulting split in mind‐brain unity had profound effects on medicine, philosophy and psychology. One might contend that this split perpetuated the dualism of Descartes and that Cartesian Dualism continues to divide concepts and fields which ultimately belong together (31).

While psychiatry and neurology proceeded along parallel but separate tracks from the 1890's, neuroscience flourished with the elaboration of principles, such as synaptic action, neurotransmission, and the chemical mediation of neuronal signaling. Only in about 1950 did it became possible to record from individual neurons in living animals (32). A conceptual framework for experimental brain–mind integration was concurrently provided by the discoveries of brain activation in waking (33) and in REM sleep dreaming (34). These discoveries resulted from the technological advance of recording the electrical activity of the brain via the electroencephalogram (EEG) (35).

These neuroscientific advances led to some important insights: The localization of REM sleep control to the pontine brain stem (36), the modulation of cerebral activation by aminergic brain stem neurons (37), the mediation of REM sleep events—including REMs themselves—and the inhibition of motor tonus by specific neurophysiological mechanisms (38). Extracellular neuronal recording produced neurophysiological data from REM sleep that was subsequently integrated with quantitative analysis of dream mentation, to create the first brain‐based theory of dreaming (20, 30). Over the subsequent four decades these findings have been confirmed and enriched (39, 40).

### Lucid Dreaming

**FIGURE 1.**
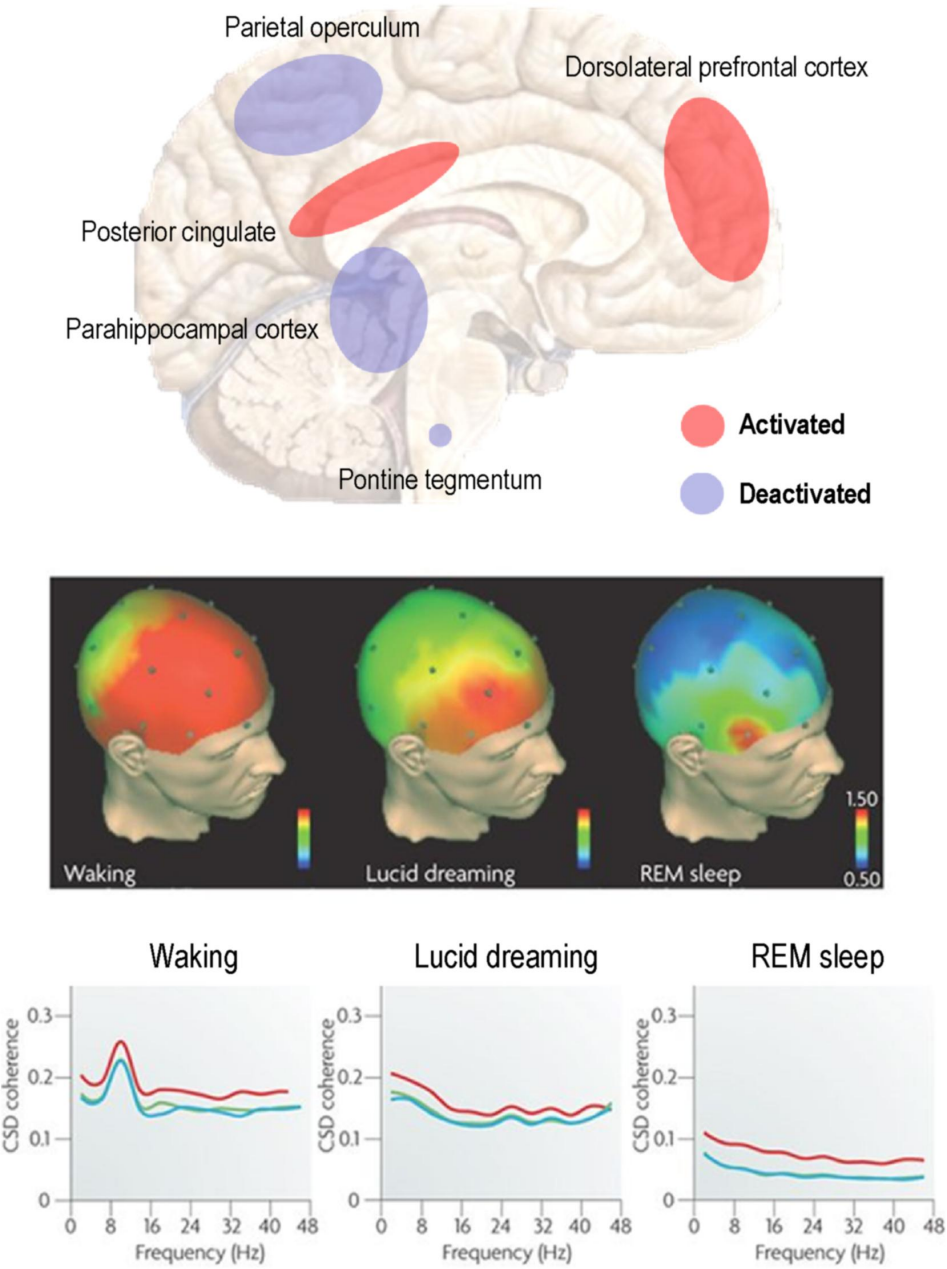
Upper panel: Blue and red areas indicate regions typically showing hyperactivation and deactivation, respectively, during REM, relative to waking, as measured by positron emission tomography. Lesions to the parietal operculum are associated with the loss of dreaming following stroke or prefrontal lobotomy. Middle panel: Quantitative electroencephalographic (EEG) studies—comparing brain activity during waking, lucid dreaming and REM sleep—suggest that certain frontal areas are highly activated during waking but show deactivation during REM sleep. During lucid dreaming there is an increase in gamma (40 Hz) power and coherence in frontal areas compared with non‐lucid REM sleep. Scale bars indicate standardized power based on scale potentials (0.50% to 1.50% power). Lower panel: In addition to the increased 40 Hz EEG power in frontal channels, EEG coherence is much higher during lucid dreaming than during non‐lucid REM sleep. EEG coherence during lucid REM sleep corresponds to that during waking (left panel); except for the 8–12 Hz alpha range that shows a peak during waking. CSD, cross‐spectral density. Adapted with permission from (13). REM, rapid eye movement

The psychologist Ursula Voss used EEG to show that when human subjects became aware that they were dreaming—instead of erroneously supposing themselves to be awake as is usual in REM sleep—they exhibited the frontal lobe activation, normally seen in waking—together with parietal and occipital signs of REM sleep (41): see Figure 1. These findings were later supported by a case study using combined EEG and functional magnetic resonance imaging (42), showing heightened BOLD activation in frontal areas, in addition to the precuneus and inferior parietal lobules; all of which are implicit in self‐referential processing and the experience of agency (43).

This finding was supported by a subsequent study showing heightened activity in the dorsolateral prefrontal cortex during dream lucidity (44) and a further study showing elevated resting‐state functional connectivity between the prefrontal and temporoparietal association areas in frequent lucid dreamers (45). Lucid dreaming thus reveals itself to be a hybrid state of the brain–mind, with both waking and dreamlike features—with its phenomenal aspects being reducible to differential activity in specific brain structures (46). This suggests the brain and the mind operate as one, with even the highest features of conscious activity—such as self‐referential processing and insight—having definitive and functionally segregated neuronal correlates.

A related point is that the brain–mind is not necessarily unified with respect to its own cardinal states. The expression “I am of two minds” reflects the ambivalence (or multivalence) of cognition (and emotion) in waking consciousness. The locality of brain activation has been clearly demonstrated in studies of regional cortical activation in NREM sleep (47) and is often referred to as “local” sleep (48, 49, 50). Normal and abnormal conscious experience may thus be not only linked in an intimate way to neuronal dynamics but show the same kind of segregation and differentiation seen in the neurophysiological correlates of sleep (47, 51, 52).

### Human REM Sleep Signaling

**FIGURE 2.**
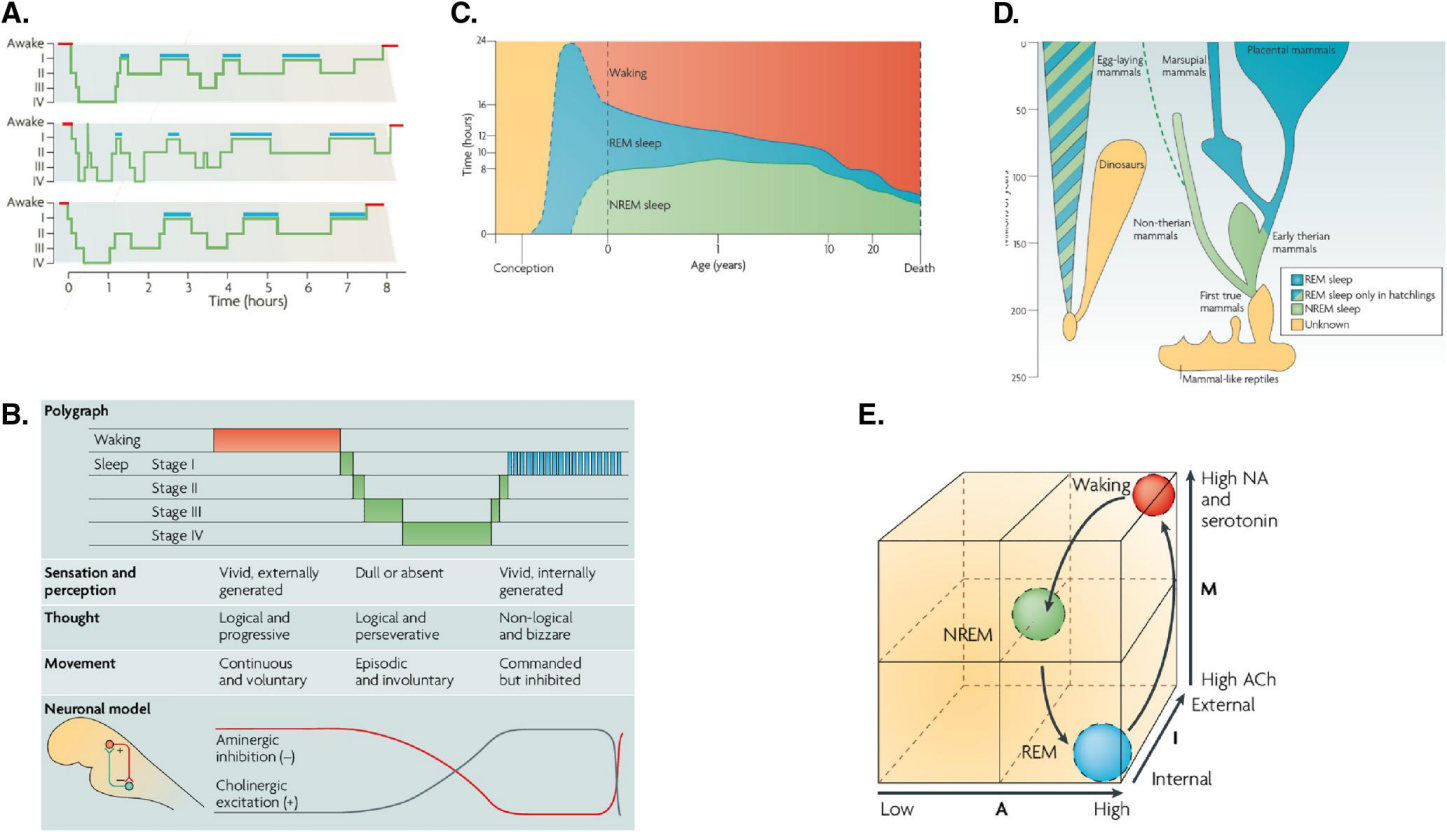
A. Standard sleep polygraphic measurements. These traces show 90−100 min cycles of rapid eye movement (REM) and non‐rapid eye movement (NREM) sleep. The traces show cycles for three subjects, where the blue lines indicate periods of REM sleep. Reports of dreaming are most common from sleep onset stage I (when dreams tend to be fragmentary), late‐night stage II (when dreams tend to be thought‐like) and REM (when they tend to be long, vivid, hallucinatory and bizarre). Deep phases of sleep (III and IV) occur in the first half of the night, whereas lighter stages (stages I and II) predominate in the second half. B. The states of waking and sleep. These states have behavioral, polygraphic and psychological correlates that appear to be orchestrated by a control system in the pontine brainstem. In this panel, the neuronal clock that controls these states is depicted as a reciprocal interaction between inhibitory aminergic neurons and excitatory cholinergic neurons: aminergic activity is highest during waking, declines during NREM sleep and is lowest during REM sleep; whereas cholinergic activity shows the reverse pattern. Changes in sleep phase occur whenever the two activity curves cross; these are also the times when major postural shifts occur. The motor immobility during sleep depends on two different mechanisms: disfacilitation during stages I−IV of NREM sleep and inhibition of motor systems during REM sleep. The motor inhibition during REM sleep prevents motor commands from being executed, so that we do not act out our dreams. C. Human sleep and age. The preponderance of rapid eye movement (REM) sleep in the last trimester of pregnancy and the first year of life decreases progressively as waking time increases. Note that NREM sleep time, like waking time, increases after birth. Despite its early decline, REM sleep continues to occupy approximately 1.5 h/day throughout life. This suggests that its strongest contribution is during neurodevelopment but that it subsequently plays an indispensable role in adulthood. D. The evolution of REM sleep. Birds and mammals evolved separately after branching off from the ancestral tree. Both birds and mammals are homeothermic, and both have appreciable cognitive competence. With respect to the enhancement of cognitive skills by REM, it is significant that both birds and mammals are capable of problem solving and both can communicate verbally. E. AIM model. This panel illustrates normal transitions within the AIM state‐space from waking to NREM and then to REM sleep. The *x*‐axis represents A (for activation), the *y*‐axis represents M (for modulation) and the *z*‐axis represents I (for input–output gating). Waking, NREM sleep and REM sleep occupy distinct loci in this space. Waking and REM sleep have high activation but different I and M values. Thus, in REM sleep, the brain is both off‐line and chemically differentiated compared with the waking brain. NREM sleep is positioned in the center of the space because it is intermediate in all quantitative respects between waking and REM sleep: adapted from (13)

The Helmholtzian pillar of the current thesis associates unconscious inference with perception and sentience (4, 6). Formal treatments of this notion rest upon predictive processing; namely, using sensory impressions to confirm or disconfirm hypotheses about how our sensory inputs are generated (53). When sensations are generated actively, the accompanying predictions must reflect the way that the sensorium is sampled. Until recently, the electrical encoding of internal predictions that accompany eye movements had only been demonstrated in cats (11, 36). The psychiatrist Charles Hong used brain imaging to demonstrate robust evidence for the existence of these internal stimuli in human REM sleep (54). Each and every human eye movement command—from the pontine brain stem to the forebrain—is associated with information transfer that assures perceptual integrity in waking. And which may be used to construct the visual imagery of dreams. Hong et al (55) have recently discussed these possibilities and how they might be pursued to establish the unity of the brain–mind in visual perception, be it external (as in waking) or internal (as in dreaming). Much of this empirical work was motivated by the AIM model of sleep which we now briefly summarize (see also Figure 2).

### The AIM Model

**FIGURE 3.**
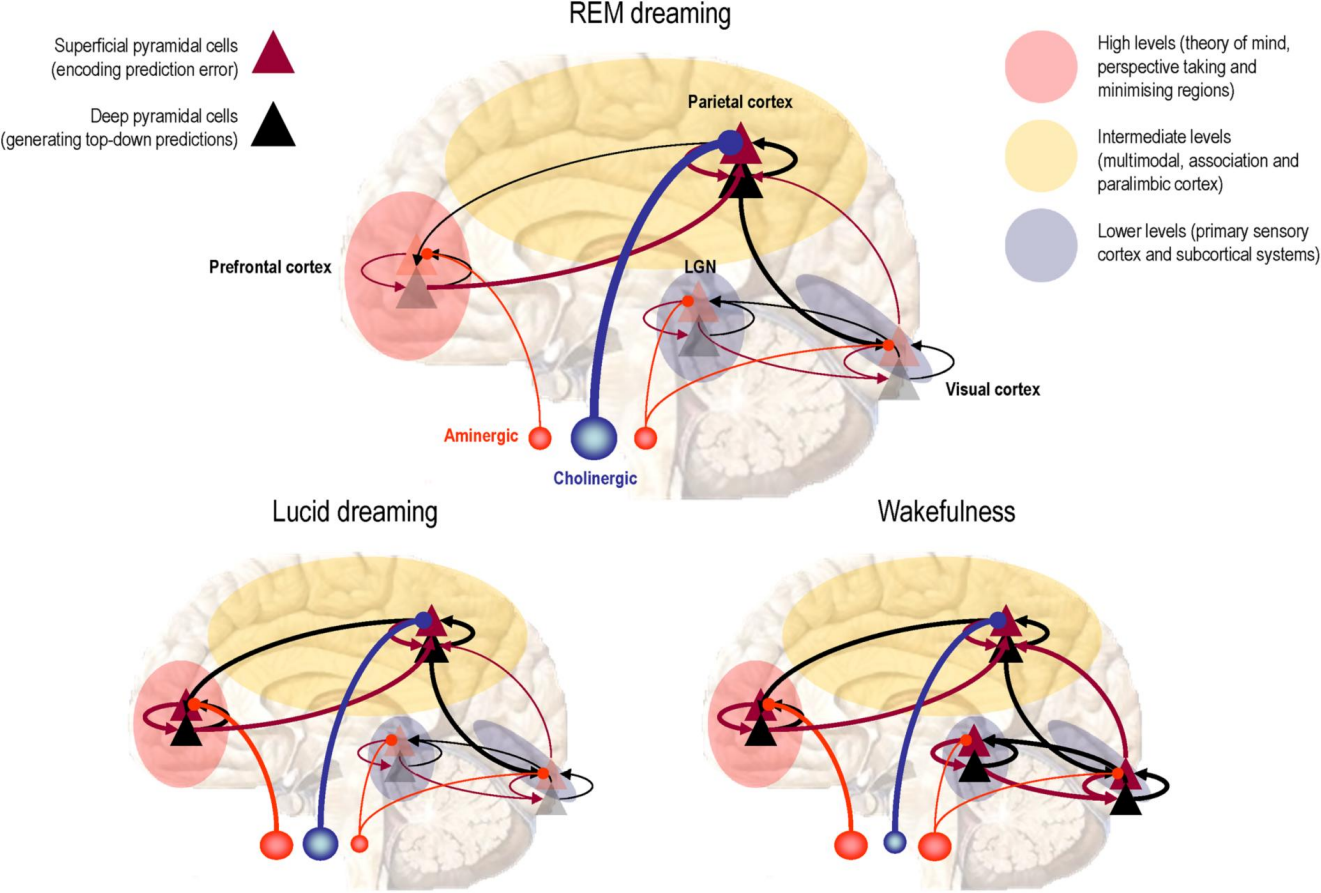
The schematic illustrates the neuromodulatory gating of hierarchical message passing in the brain during REM sleep (top panel), wakefulness (right panel) and the hybrid state of lucid dreaming (left panel). The anatomy of this schematic should not be taken too seriously: it is just meant to differentiate between different levels of the cortical hierarchy in terms of low (sensory) levels, intermediate (extrasensory and multimodal) levels and—for the purposes of this essay—high (meta‐representational) levels. Here, we have associated higher levels with theory of mind areas that are engaged in mentalising and perspective taking (and are a component of the default mode). Within each level we have depicted representative cortical microcircuits in terms of superficial (red triangles) and deep (black triangles) pyramidal cells. In predictive coding formulations of neuronal message passing, superficial pyramidal cells encode prediction error that is passed up the hierarchy to update the activity of deep pyramidal cells encoding expectations (red connections). These reciprocate top‐down predictions—that are compared with expectations—by prediction error units in the level below (black connections). In REM sleep, the idea is that cholinergic modulation (blue projections) of superficial pyramidal cells at intermediate levels of the cortical hierarchy preferentially enables these levels, while suppressing ascending prediction errors from primary sensory cortex. In lucid dreaming, aminergic (pink projections) neuromodulation sensitizes prediction errors in the prefrontal cortex, enabling top‐down predictions from the highest deepest levels of the hierarchy—endowing processing in intermediate levels with a narrative or context. In waking, aminergic (e.g., noradrenergic) neuromodulation boosts sensory prediction errors that are now able to entrain hierarchical inference in higher cortical levels for perceptual synthesis. Modified with permission from (62). REM, rapid eye movement

The activation‐synthesis hypothesis of dreaming (20, 21, 30) and the reciprocal interaction model of sleep cycle control (20) have been modified, simplified and extended to accommodate altered states of consciousness: see Figure 2 and (13). The ensuing AIM account tries to integrate neurophysiology and psychology as follows. The three dimensions of AIM state‐space represent activation (A), input source (I) and modulation (M), as measured neurophysiologically. The discovery of REM‐locked pontine activity in humans (54) enriched the AIM model by providing evidence for human ponto‐geniculo‐occipital (PGO) wave activity (56) that had only been definitively recorded in the pons (57). Figure 3 provides a schematic based on the AIM model in Figure 2 that focuses on the functional anatomy of sleep in terms of neuromodulation. This account is scaffolded on functional brain states and neuromodulation at the level of synaptic physiology, which brings us to the psychopharmacology of sleep—and its intersection with psychopathology.

The aminergic and cholinergic neurons that underwrite conscious states are affected by drugs of common use in medicine. These include atropine and the antidepressants (especially the amine reuptake blockers). Because such drugs have both short and long term effects they are useful as sedatives and mood regulators, depending on dose; for example, (58). This is not surprising given the well‐known intimacy of sleep and affect, where the most prominent psychiatric beneficiary of sleep science is mood disorder (59). The reduction in REM sleep latency seen in depression predicts positive response to amine reuptake treatment (60, 61). This speaks to the role of classical ascending modulatory neurotransmitters in the control of both sleep and mood. In the next section, we take some of the above fundaments of sleep and dream science and see how they relate to formulations of the sentient brain, in terms of predictive processing and belief updating. We will see that neuromodulation is a recurring theme that may have a special relevance for psychiatry.

## The Phantastic Organ

In this section, we integrate the above themes to show how they underwrite a mechanics of sentience that affords a mechanistic (sic) account of psychopathology and pathophysiology. This account is coarse‐grained but highlights the cross‐cutting themes that link a diverse range of psychiatric and neurological conditions. Our starting point is a commitment to the mind as a brain‐based process; namely, the process of belief updating (6). In this setting, beliefs are used in the sense of Bayesian beliefs or probabilistic representations (7, 8, 63). These are largely of a subpersonal sort, as opposed to propositional beliefs. A central tenet of this formulation of sentience is that it accounts for previous experience (so‐called priors) in the prediction of what causes sensations and consequent behavior.

On this view, mindful processes can be described as inference; that is, inferring the causes of sensations—and indeed acting to elicit sensory information to enable inference. This is a key move in two respects: first, it is necessary when talking about the symptoms, signs and experience of patients, whose phenomenology is quintessentially belief‐based. In other words, nearly all psychiatric syndromes can be framed as aberrant belief updating or false inference. Obvious examples here include hallucinations and delusions; namely, inferring things are there, when they are not (25, 64). Conversely, various forms of agnosia and dissociative syndromes speak to an inference about things that are not present when they are (65, 66). On a statistical reading, these are just two facets of standard inference; referred to as type I and II errors, respectively. The second reason for framing things around inference draws on fluctuating states of consciousness and, in particular, dreaming. If one wanted definitive evidence—phenomenal, physiological, or philosophical—for the brain as a Helmholtz machine or statistical organ, one need look no further than the phenomena of dreaming (3, 19, 24, 40, 67, 68). Dreams evince the remarkable capacity of our brains to generate and construct worlds, even when sequestered from the sensorium, via carefully orchestrated neuromodulatory disconnections (see Figure 3).

The basic idea here is that the brain is a phantastic organ, generating phantasies that it puts to the test—in waking—by using sensory samples from the world (69). However, in dreaming, the fidelity or precision of these sensory inputs is attenuated, enabling a virtual reality to play out in our heads—a process that has been formalized in terms of Bayesian model reduction and complexity minimization (19). The theme of the brain as an organ of prediction now dominates much of cognitive neuroscience in the form of active inference or predictive processing (8, 70, 71, 72). Active inference brings an important (and enactive) aspect to inference; namely, the fact that we actively gather the information upon which inference is based (73). In the current setting, this becomes important when we consider what false inference might look like in an enactive setting. It transpires that several neurological conditions then come under the same explanatory compass; these include Parkinson's disease and other movement disorders associated with synaptopathies (66, 74). To understand this, one has to drill down on a fundamental feature of inference; namely, the encoding or quantification of uncertainty, which we will consider in terms of precision (75, 76, 77).

### Precision Psychiatry

There are several ways in which we can license a focus on precision and the encoding of uncertainty in active inference. We will briefly rehearse a few prescient approaches, to show that they all converge on the same conclusion; namely, inference—and especially *false inference*—depends sensitively on getting precision right (78). Getting precision right depends upon the right deployment of neuromodulatory mechanisms that coordinate message passing in cortical and subcortical hierarchies (76, 79).

First, from a purely technical perspective, a commitment to a dual aspect monism requires the existence of a dual aspect information geometry that links neuronal dynamics to belief updating (10). The technical details are beyond the scope of this essay; however, they can be understood heuristically in terms of two geometries that describe the statistics of neuronal activity (and connectivity) on the one hand, and statistics of (Bayesian) beliefs encoded by that activity (and connectivity) on the other. In other words, there are two information geometries that inherit from the ensemble dynamics of neuronal populations. The first pertains to the probabilistic evolution of neuronal states, while the second pertains to the evolution (i.e., updating) of beliefs *about something*—beliefs that are encoded by neuronal states (and connectivity). Crucially, these two information geometries arise in any system that self‐organizes itself in a way that can be separated from its environment (80, 81, 82).

This may sound rather abstract; however, it is just a statement of a fact—for which we are our own existence proof. Namely, the neurophysiology and neurochemistry of our brains conforms to the laws of thermodynamics and an associated information geometry (83). At the same time, these physiological states (c.f., the AIM model) are necessarily equipped with an information geometry that pertains to things and narratives “out there.” Dreaming is a beautiful example of this: my brain enters a particular sleep (e.g., REM) state, characterized by a particular physiology, while at the same time I dream *about* things in my lived world. Crucially, the things I dream about are essentially grounded in my navigation of that world. In other words, REM dreams are always *animated* (hence in the service of running motor programs offline), *emotional* (with anxiety, aggression, and elation predominating), *bizarre* (meaning only remotely associative) and so on (13, 84). “Chase” dreams are common and well‐known, and frequently overlap with—and merge—into nightmares. In the dream state, one rehearses threatening situations (running away, turning, and fighting) but also simulates more abstract potentialities. One can fly or make love (85), particularly when lucid (86).

### An Intuitive Physics for the Mind

Technically, information geometries underwrite a (Bayesian) mechanics of the self‐organizing brain and thereby characterize the nature of belief spaces (82). This can be appreciated from a number of complementary perspectives. For example, one can apply gauge theoretic treatments (87). Alternatively, one can think about the notion of distance and information length—as measures of how far beliefs move during inference or belief updating (83, 88). All these treatments converge on the same quantity; namely, the precision of beliefs—that is, the confidence or inverse variance of a belief distribution.

Information geometry allows us to conceive of belief‐spaces in which two beliefs are equipped with a measure of distance between them. The key notion here is some state‐space (e.g., the physiological states of the AIM model), where every point in this space corresponds to a Bayesian belief (i.e., a probability distribution). This special sort of state‐space is called a statistical manifold. For example, imagine I saw something fluttering out of the corner of my eye. On foveating the source of “fluttering,” I “see” it is a butterfly. This active sampling of the visual scene moves my (prior) beliefs that there was a small creature out there—with probability mass distributed over all kinds of plausible alternatives (e.g., insect, bird, leaf, etc.)—to a precise (posterior) belief (e.g., butterfly). This belief updating corresponds to moving from one point on a statistical manifold to another. So, what are the key determinants of that movement and how is movement on a statistical manifold measured?

In statistics, this measure is based on the Fisher information metric. Under mild (i.e., Gaussian) assumptions, this metric corresponds to the precision or confidence associated with (probabilistic or posterior) beliefs. Intuitively, we can envisage a myriad of beliefs occupying some belief space, all attracting each other to a greater or lesser degree. This is very much like a celestial *n*‐body problem, in which heavenly bodies, pull each other in different directions—to find their minimum free energy configuration. In this intuitive physics, precision corresponds to the mass of each belief (or associated prediction error). In other words, a belief (or associated prediction error) that is afforded greater precision will behave like a massive body—attracting smaller beliefs to it—and becoming relatively impervious to others. For example, if *sensory* precision were inordinately high, believes based upon sensory evidence would acquire a gravity that pull other (empirical prior) beliefs towards it. This attraction is the manifestation of (mindful) forces that underwrite belief updating from *prior* to *posterior* Bayesian beliefs, that is, before and after the effects of sensory forces. Conversely, if prior precision is high the forces exerted by sensory impressions exert less influence on beliefs deep within a hierarchical belief system. These two extremes are just the difference between waking and sleep, respectively; in waking we have information from the outside world to shape perception, whereas in sleep, we are sequestered from these sensory impressions.

On this view, one can see how getting precision right becomes crucial: precision is the glue, or bridge that realizes conditional dependencies among distinct beliefs about the lived world. If this coupling were to fail, there would literally be a disintegration of the psyche and loss of central (deep) coherence that characterizes conditions like schizophrenia and autism (89, 90, 91, 92). Indeed, as we will see below, any form of belief updating must have precision at its heart, suggesting that psychopathology is a pathology of precision.

### Predictive Processing and Precision

From the perspective of predictive coding (a canonical scheme for predictive processing) (93, 94), precision is the key quantity that coordinates belief updating by differentially weighting prediction errors as they ascend cortical hierarchies (75, 95, 96, 97, 98, 99). In these schemes, prediction errors represent the difference between sensory input and top‐down predictions of that input. Any mismatch then revises Bayesian beliefs or expectations encoded by neuronal activity to implement belief updating—and provide better predictions that resolve prediction errors (see Figure 3). In engineering, precision is known as Kalman gain (100) and scores the precision or reliability of prediction errors; such that only precise prediction errors have access to higher levels of belief updating (101). In this setting, it can be seen that getting the precision or gain right is crucial for veridical inference.

From a physiological perspective, precision is thought to be encoded in the synaptic gain or excitability of particular neuronal populations (e.g., superficial pyramidal cells encoding prediction errors). It can be subsumed under notions of excitation‐inhibition balance or cortical gain control at the population level (102, 103). At the synaptic level, it encompasses a broad range of neuromodulatory mechanisms that generally involve fast‐spiking inhibitory interneurons that Interact with pyramidal cells (104, 105, 106, 107, 108). In turn, these (often synchronous) interactions depend upon N‐methyl‐D‐aspartate (NMDA) receptor function and, crucially, classical ascending modulatory neurotransmitter systems (e.g., noradrenaline, serotonin, and acetylcholine) (109, 110).

From a psychological perspective, precision manifests as various forms of attentional gain control or selection (101, 111). The right deployment of sensory precision in the visual domain can be thought of as mediating spatial attention, whereas higher in the hierarchy it can be associated with feature attention and, possibly, internal attention states (112, 113).

It is at this point that we see the formal relationship between precision and the AIM model (21). The activation (A) of certain neuronal populations—that entail belief updating—depends upon their input (I), which is under modulatory control (M). The permissive requirements for sleep rest upon a selective disconnection of sensory inputs via aminergic neuromodulation, which can be thought of as a physiologically mediated inattention to the sensorium. In many respects, it is the exact complement of mindfulness, in which sustained attention to a specific sensory input is maintained (114).

Finally, from a more philosophical perspective, the hierarchical control or predictions of precision underwrite notions of mental action and the distinction between the phenomenal transparency and capacity (68, 115, 116, 117). When associated with interoceptive inference, high level representations—that themselves predict the precision of lower‐level representations—may have a crucial role in emotional inference and elaborating a minimal selfhood (98, 115, 118).

In summary, a ubiquitous and key aspect of belief updating, or active inference, is the mechanics of encoding uncertainty, by optimizing (Bayesian) beliefs *about precision*. Mechanistically, this speaks to a key role of neuromodulation and the context‐sensitive control of synaptic efficacy. Psychologically, it ties in attention to any consideration of mindful processing (i.e., inference). Finally, these mechanisms are evinced most powerfully by the greatest contrast of sentience we know; namely, the difference between waking perception and dreaming. We now turn to aberrant precision in neuropsychiatry.

### Aberrant Precision

In the past years, nearly every psychiatric condition has been considered under the lens of precision; ranging from autism through to schizophrenia; from tremor through to Parkinson's disease; from obsessive‐compulsive disorder through to post‐traumatic stress disorder; from depression through to chronic stress; from fatigue through to functional medical symptoms (65, 97, 119, 120, 121, 122, 123, 124, 125, 126, 127, 128, 129, 130). Table 1 provides a selective survey of some key publications, all pivoting on aberrant precision control. Each entry in Table 1 deserves its own discussion, showing how the same fundamental deficit in aberrant precision or attentional processing can manifest in domains as diverse as motor control to depersonalization. In what follows, we will focus on a couple of cardinal examples.

**TABLE 1:** A selection of recent papers dealing with predictive coding, precision and psychiatry

Syndrome symptom	Selected papers
Precision, predictive coding and Bayesian inference in schizorphenia	Pollak TA, Corlett PR. Blindness, psychosis, and the visual Construction of the world. *Schizophr Bull*. 2019 Oct 11. pii: sbz098. doi: https://doi.org/10.1093/schbul/sbz098. [Epub ahead of print]
	Benrimoh et al. (2019) (135)
	Sterzer et al. (2018) (158)
	Dzafic I, Burianová H, Martin AK, Mowry B. Neural correlates of dynamic emotion perception in schizophrenia and the influence of prior expectations. *Schizophr Res*. 2018 Dec; 202:129–137
	Heinz A, Murray GK, Schlagenhauf F, Sterzer P, Grace AA, Waltz JA. Towards a Unifying cognitive, neurophysiological, and computational neuroscience account of schizophrenia. *Schizophr Bull*. 2019 Sep 11; 45 (5):1092–1100
	Limongi R, Bohaterewicz B, Nowicka M, Plewka A, Friston KJ. Knowing when to stop: Aberrant precision and evidence accumulation in schizophrenia. *Schizophr Res*. 2018 Jul; 197:386–391
	Randeniya R, Oestreich LKL, Garrido MI. Sensory prediction errors in the continuum of psychosis. *Schizophr Res*. 2017 Apr 27. pii: S0920‐9964 (17)30206‐2
	Griffin JD, Fletcher PC. Predictive processing, source monitoring, and psychosis. *Annu Rev Clin Psychol*. 2017 May 8; 13:265–289
	Corlett PR. I predict, Therefore I Am: Perturbed predictive coding under ketamine and in schizophrenia. *Biol Psychiatry*. 2017 Mar 15; 81 (6):465–466
	Tschacher W, Giersch A, Friston K. Embodiment and schizophrenia: A review of implications and applications. *Schizophr Bull*. 2017 Mar 3
	van Schalkwyk GI, Volkmar FR, Corlett PR. A predictive coding account of psychotic symptoms in autism spectrum disorder. *J Autism Dev Disord*. 2017 May; 47 (5):1323–1340
	Schmack K, Rothkirch M, Priller J, Sterzer P. Enhanced predictive signalling in schizophrenia. *Hum Brain Mapp*. 2017 Apr; 38 (4):1767–1779
	Sterzer P, Mishara AL, Voss M, Heinz A. Thought Insertion as a self‐Disturbance: An integration of predictive coding and Phenomenological approaches. *Front Hum Neurosci*. 2016 Oct 12; 10:502. eCollection 2016
	Kort NS, Ford JM, Roach BJ, Gunduz‐Bruce H, Krystal JH, Jaeger J, Reinhart RM, Mathalon DH. Role of N‐Methyl‐D‐Aspartate receptors in action‐based predictive coding deficits in schizophrenia. *Biol Psychiatry*. 2017 Mar 15; 81 (6):514–524
	Friston K, Brown HR, Siemerkus J, Stephan KE. The dysconnection hypothesis (2016). *Schizophr Res*. 2016 Oct; 176 (2‐3):83–94
	Roa Romero Y, Keil J, Balz J, Gallinat J, Senkowski D. Reduced frontal theta oscillations indicate altered crossmodal prediction error processing in schizophrenia. *J Neurophysiol*. 2016 Sep 1; 116 (3):1396–1407
	Wacongne C. A predictive coding account of MMN reduction in schizophrenia. *Biol Psychol*. 2016 Apr; 116:68–74
	Powers et al. (2015) (136)
	Adams RA, Huys QJ, Roiser JP. Computational psychiatry: Towards a mathematically informed understanding of mental illness. *J Neurol Neurosurg Psychiatry*. 2016 Jan; 87 (1):53–63
	Rentzsch J, Shen C, Jockers‐Scherübl MC, Gallinat J, Neuhaus AH. Auditory mismatch negativity and repetition suppression deficits in schizophrenia explained by irregular computation of prediction error. *PLoS One*. 2015 May 8; 10 (5):e0126775
	Castelnovo A, Ferrarelli F, D'Agostino A. Schizophrenia: From neurophysiological abnormalities to clinical symptoms. *Front Psychol*. 2015 Apr 20; 6:478
	Notredame et al. (2014) (137)
	Fogelson et al. (2014) (149)
	Horga G, Schatz KC, Abi‐Dargham A, Peterson BS. Deficits in predictive coding underlie hallucinations in schizophrenia. *J Neurosci*. 2014 Jun 11; 34 (24):8072–8082
	Jardri R, Denève S. Circular inferences in schizophrenia. *Brain*. 2013 Nov; 136(Pt 11):3227–3241
	Ford JM, Palzes VA, Roach BJ, Mathalon DH. Did I do that? Abnormal predictive processes in schizophrenia when button pressing to deliver a tone. *Schizophr Bull*. 2014 Jul; 40 (4):804–812
	Adams et al. (2013) (138)
	Nazimek JM, Hunter MD, Woodruff PW. Auditory hallucinations: Expectation‐perception model. *Med Hypotheses*. 2012 Jun; 78 (6):802–810
	Lalanne L, van Assche M, Giersch A. When predictive mechanisms go wrong: Disordered visual synchrony thresholds in schizophrenia. *Schizophr Bull*. 2012 May; 38 (3):506–513
Precision, predictive coding and Bayesian inference in autism and autistic spectrum disorder	Lanillos P, Oliva D, Philippsen A, Yamashita Y, Nagai Y, Cheng G. A review on neural network models of schizophrenia and autism spectrum disorder. *Neural Netw*. 2019 Nov 13; 122:338–363
	Van de Cruys S, Perrykkad K, Hohwy J. Explaining hyper‐sensitivity and hypo‐responsivity in autism with a common predictive coding‐based mechanism. *Cogn Neurosci*. 2019 Jul; 10 (3):164–166
	Lawson et al. (2017) (133)
	Chambon V, Farrer C, Pacherie E, Jacquet PO, Leboyer M, Zalla T. Reduced sensitivity to social priors during action prediction in adults with autism spectrum disorders. *Cognition*. 2017 Mar; 160:17–26
	van Schalkwyk GI, Volkmar FR, Corlett PR. A predictive coding account of psychotic symptoms in autism spectrum disorder. *J Autism Dev Disord*. 2017 May; 47 (5):1323–1340
	Van de Cruys S, Van der Hallen R, Wagemans J. Disentangling signal and noise in autism spectrum disorder. *Brain Cogn*. 2017 Mar; 112:78–83
	Manning C, Kilner J, Neil L, Karaminis T, Pellicano E. Children on the autism spectrum update their behaviour in response to a volatile environment. *Dev Sci*. 2016 Aug 6. doi: https://doi.org/10.1111/desc.12435
	Chan JS, Langer A, Kaiser J. Temporal integration of multisensory stimuli in autism spectrum disorder: a Predictive coding perspective. *J Neural Transm (Vienna)*. 2016 Aug; 123 (8):917–923
	von der Lühe T, Manera V, Barisic I, Becchio C, Vogeley K, Schilbach L. Interpersonal predictive coding, not action perception, is impaired in autism. *Philos Trans R Soc Lond B Biol Sci*. 2016 May 5; 371 (1693)
	Gonzalez‐Gadea ML, Chennu S, Bekinschtein TA, Rattazzi A, Beraudi A, Tripicchio P, Moyano B, Soffita Y, Steinberg L, Adolfi F, Sigman M, Marino J, Manes F, Ibanez A. Predictive coding in autism spectrum disorder and attention deficit hyperactivity disorder. *J Neurophysiol*. 2015 Nov; 114 (5):2625–2636
	Palmer CJ, Seth AK, Hohwy J. The felt presence of other minds: Predictive processing, counterfactual predictions, and mentalising in autism. *Conscious Cogn*. 2015 Nov; 36:376–389
Precision, predictive coding and Bayesian inference in depression, stress and anxiety	Linson A, Parr T, Friston KJ. Active inference, stressors, and psychological trauma: A neuroethological model of (mal)adaptive explore‐exploit dynamics in ecological context. *Behav Brain Res*. 2019 Dec 9; 380:112421
	Kube T, Schwarting R, Rozenkrantz L, Glombiewski JA, Rief W. Distorted cognitive processes in major depression: A predictive processing perspective. *Biol Psychiatry*. 2019 Jul 29. pii: S0006‐3223 (19)31550‐1
	Adams RA, Huys QJ, Roiser JP. Computational psychiatry: Towards a mathematically informed understanding of mental illness. *J Neurol Neurosurg Psychiatry*. 2016 Jan; 87 (1):53–63
	Clark et al. (2018) (128)
	Adam Linson, Karl Friston. Reframing PTSD for computational psychiatry with the active inference framework. *Cogn Neuropsychiatry*. 2019; 24 (5): 347–368
	Barrett LF, Quigley KS, Hamilton P. An active inference theory of allostasis and interoception in depression. *Philos Trans R Soc Lond B Biol Sci*. 2016 Nov 19; 371 (1708). pii: 20160011
	Seth and Friston (2016) (97)
	Stephan et al. (2016) (129)
	Schutter DJ. A Cerebellar framework for predictive coding and Homeostatic Regulation in depressive disorder. *Cerebellum*. 2016 Feb; 15 (1):30–33
	Chekroud (2015) (168)
	Cornwell et al. (2017) (127)
	Kim MJ, Shin J, Taylor JM, Mattek AM, Chavez SJ, Whalen PJ. Intolerance of uncertainty predicts increased Striatal volume. *Emotion*. 2017 May 18
	Trapp S, Kotz SA. Predicting Affective information ‐ an evaluation of Repetition Suppression effects. *Front Psychol*. 2016 Sep 9; 7:1365
	Garfinkel SN, Seth AK, Barrett AB, Suzuki K, Critchley HD. Knowing your own heart: Distinguishing interoceptive accuracy from interoceptive awareness. *Biol Psychol*. 2015 Jan; 104:65–74
	Lawson RP, Rees G, Friston KJ. An aberrant precision account of autism. *Front Hum Neurosci*. 2014 May 14; 8:302
Precision, predictive coding and Bayesian inference in hallucinations and hallucinosis	Powers AR, Corlett PR, Ross DA. Guided by Voices: Hallucinations and the psychosis spectrum. *Biol Psychiatry*. 2018 Sep 15; 84 (6):e43–e45
	Powers et al. (2017) (64)
	Corlett PR, Horga G, Fletcher PC, Alderson‐day B, Schmack K, powers AR 3rd. Hallucinations and Strong priors. *Trends Cogn Sci*. 2019 Feb; 23 (2):114–127
	Sterzer et al. (2018) (158)
	O'Callaghan C, Hall JM, Tomassini A, Muller AJ, Walpola IC, Moustafa AA, Shine JM, Lewis SJG.Visual hallucinations are characterized by Impaired sensory evidence Accumulation: Insights from hierarchical Drift Diffusion modeling in Parkinson's disease. *Biol Psychiatry Cogn Neurosci Neuroimaging*. 2017 Nov; 2 (8):680–688
	Sterzer P, Mishara AL, Voss M, Heinz A. Thought Insertion as a self‐Disturbance: An integration of predictive coding and Phenomenological approaches. *Front Hum Neurosci*. 2016 Oct 12; 10:502
	Powers AR III, Kelley M, Corlett PR. Hallucinations as top‐down effects on perception. *Biol Psychiatry Cogn Neurosci Neuroimaging*. 2016 Sep; 1 (5):393–400
	Griffin JD, Fletcher PC. Predictive processing, source monitoring, and psychosis. *Annu Rev Clin Psychol*. 2017 May 8; 13:265–289
	Schmack K, Rothkirch M, Priller J, Sterzer P. Enhanced predictive signalling in schizophrenia. *Hum Brain Mapp*. 2017 Apr; 38 (4):1767–1779
	Sterzer P, Mishara AL, Voss M, Heinz A. Thought Insertion as a self‐Disturbance: An integration of predictive coding and Phenomenological approaches. *Front Hum Neurosci*. 2016 Oct 12; 10:502
	Roa Romero Y, Keil J, Balz J, Gallinat J, Senkowski D. Reduced frontal theta oscillations indicate altered crossmodal prediction error processing in schizophrenia. *J Neurophysiol*. 2016 Sep 1; 116 (3):1396–1407
	Teufel C, Subramaniam N, Dobler V, Perez J, Finnemann J, Mehta PR, Goodyer IM, Fletcher PC. Shift toward prior knowledge confers a perceptual advantage in early psychosis and psychosis‐prone healthy individuals. *Proc Natl Acad Sci U S A*. 2015 Oct 27; 112 (43):13401–13406
	Powers et al. (2015) (136)

The thesis developed here helps clarify why the AIM model translates so gracefully when describing psychopathology and other disorders of movement and perception. This follows from the simple observation that if psychology (i.e., the mind) and physiology (i.e., the brain) inherit from the same (Bayesian) mechanics, it follows that psychopathology must be a pathology of inference. The particular kind of false inference—implied by the key role of precision—is then grounded in synaptic gain control that selects the sensory inputs (and, in predictive coding, ascending prediction errors throughout cortical hierarchies) responsible for inference or belief updating. This immediately suggests that any pathophysiology that can be framed as a synaptopathy, which interferes with synaptic gain control, can be understood in terms of aberrant precision control (131). We will focus on a couple of canonical examples to illustrate the crosscutting themes.

Perhaps the poster child for this line of thinking is autism, in which the belief updating has been proposed to rest on an imbalance between sensory evidence and prior beliefs—mediated by their relative precision (91, 132, 133, 134). The synaptic mechanisms that underwrite this imbalance are much less well developed but a compelling story can still be told at the level of psychopathology. Schizophrenia, has, arguably, the more advanced story—of abnormal synaptic gain control—to accompany the concomitant failures of belief updating and inference (64, 125, 135, 136, 137, 138, 139, 140). As with many psychiatric syndromes, the story starts with a failure of sensory attenuation—in the sense of a failure to modulate sensory precision (141, 142, 143, 144, 145, 146, 147). This explains some key soft neurological signs in schizophrenia such as an attenuation of the mismatch negativity, failures of slow pursuit eye movements and failures of sensory attenuation that underlie the force matching illusion (138).

In many instances, these examples demonstrate a paradoxical veracity of perception that renders it difficult to elicit illusions in schizophrenia (137, 148). The evidence for this kind of (paradoxical) deficit usually rests upon a careful use of psychophysics and computational modeling to illustrate aberrant precision control; for example (149). The physiological concomitants are easily articulated in terms of neuronal processing (e.g., abnormal excitation‐inhibition balance) (150), right down to the receptors that may mediate this control (e.g., NMDA receptors and GABAergic neurotransmission) (103, 110, 136, 151, 152, 153, 154, 155, 156, 157, 158). In this sense, schizophrenia may be a paradigm example of a (synaptic) disconnection syndrome that is largely manifest in terms of abnormal perception and belief updating (i.e., hallucinations and delusions). If this is the right story, then one would expect to see very similar phenomenology in other conditions that compromise synaptic gain control. Key examples here are synaptopathies; for example, Lewy body disease leading to hallucinosis (25, 159).

One key question in schizophrenia research, in this setting, is the emergence of delusions—which at first glance seem a prime candidate for high‐level belief systems that are afforded “too much” precision. This appears to fly in the face of a failure of sensory attenuation (i.e., too much sensory precision). Although consensus has not yet emerged, this might be an interesting example of a (perceptual) paradoxical lesion (160, 161, 162). In other words, a synaptopathy at higher levels of cortical (hierarchical) processing that partially reverses the effects of a primary insult at the sensory level. In other words, a compensatory increase in precision of higher level belief systems may be necessary to balance a loss of attenuation at lower levels; thereby engendering delusional belief systems as the best Bayesian explanation for sensory evidence—that one has lost the capacity to ignore. This clearly has close relationships with earlier notions, such as the aberrant salience hypothesis (163, 164) and fits nicely with the effects of psychedelic and psychomimetic drugs (165).

From a practical point of view, the foregoing suggests that pharmacotherapy—targeting neuromodulatory systems—is exactly the right way to proceed. Having said this, the multiple factors involved in synaptic gain control make psychopharmacology a challenging avenue to remediate a loss of delicate balance in hierarchical neuronal message passing. On a more positive note, if one subscribes to the above story, then the top‐down control of precision weighting becomes another therapeutic target. In this sense, it speaks to the possibility of using psychotherapy in conjunction with psychopharmacology (166, 167, 168), to improve a patient's ability to orchestrate the many facets of attention.

In concluding this section, we briefly consider movement disorders. The link between movement disorders and false inference is mandated by active inference accounts of how we sample our world. There are many examples here; for example, the difficulties schizophrenic patients have with slow pursuit eye movements (125, 169). Perhaps a more fanciful example would be Parkinson's disease. However, the inferential (mindful) mechanics could be exactly the same as seen in other conditions ranging from alpha synucleinopathies (76, 158, 170) through to autonomic dysfunction (123, 171).

The idea here is that our motor and autonomic reflexes are just in the service of fulfilling top‐down predictions (97). This tenet of active inference inherits from ideomotor theories and 20th‐century formulations such as equilibrium point hypothesis (172) and perceptual control theory and the passive movement paradigm (173, 174). In brief, if all our (motor and autonomic) actions are realizations of descending (proprioceptive and interoceptive) predictions, then the precision afforded descending predictions, relative to the peripheral prediction errors that drive reflexes, becomes crucial. Indeed, Parkinson's disease could be regarded as a complete failure to attenuate proprioceptive precision, such that the intention to move is immediately subverted on the basis of (unattenuated) proprioceptive evidence one is not moving (138). The neuromodulatory correlates—on an active inference reading of motor control—of this putative failure are well characterized in terms of dopamine neurotransmission (175)—and, indeed, the electrophysiological correlates of precision control such as beta activity (176).

### Summary

In summary, a broad range of psychiatric and neurological disorders may yield to a coarse‐grained but mechanistic explanation in terms of aberrant precision control. Crucially, the mechanics at hand are of two sorts. On the one hand, there is the Bayesian mechanics of belief updating, which underwrites our perceptions and experience of the world—and how it is actively sampled—on the other hand, the synaptic mechanisms are, at a physiological, computational and population level, clearly situated in terms of their role in belief updating. In short, the encoding of uncertainty or precision in the brain inherits from a dual aspect monism.

## Discussion

In the foregoing, we have appealed to the fundaments of sleep research; both in terms of its implications for sentience and its neurophysiology to argue for a holistic framing of neuropsychiatric disorders that dissolves Cartesian dualism by treating mindful processes (i.e., belief updating) and neuronal processes (i.e., synaptic physiology) as two sides of the same coin. One could of course consider disorders cortical gain (i.e., precision) control and, indeed, sleep per se within this framework.

A particularly instructive example is the rich history of seizure disorder. Excitability is an intrinsic and essential aspect of neuronal electrical function (103, 150, 177, 178, 179), but it also constitutes a natural problem because that excitability must be controlled (180)—and this control has been considered in relation to precision (181). The brain–mind is particularly vulnerable to escape from excitability control (71). Epileptic seizures are the result when local foci of seizure discharge become generalized. The pathophysiology of epilepsy and the normal occurrence of paroxysmal neuronal firing in REM sleep have been detailed elsewhere (182). Here, we focus on two related themes: temporal lobe epilepsy and normal dreaming.

In temporal lobe epilepsy, a hyperexcitable focus arises in the limbic brain and spreads so as to take over waking consciousness. An affected subject becomes suspicious to the point of paranoia, convinced that others are talking about them, and harboring delusions of self‐importance. Great writers like Fyodor Dostoyevsky may have had hyperexcitable temporal lobes which drove their creative, and some would say, “hypergraphic” literary productivity (183).

A more physiological temporal lobe stimulation occurs in REM sleep dreaming. Four or five times a night the brainstem sends excitatory signals to the temporal lobe such that we see, hear and feel sensations and emotions not entirely unlike those of the epileptic patient. Are we to suppose that dreaming is pathological? No, but we might consider the kinship of our normal brain–mind state to the psychosis of people we call patients. Our understanding of their hallucinations and delusions makes discussion of these so‐called symptoms more direct and naturalistic. “Your visions are like my dreams” we might say. This statement is more than reassuring.

Abundant neurophysiological findings of REM abnormalities in psychiatric patients (184, 185, 186), further support the notion that psychopathology and inference have a common grounding in neuromodulatory control that transcends waking and sleeping. Furthermore, evidence of NREM sleep alterations, including deficits in sleep spindles and slow waves, have been increasingly reported in neuropsychiatric disorders, especially in patients with schizophrenia (185, 187, 188, 189, 190, 191), implicating these electrophysiological correlates of sleep in the pathophysiology of these disorders; particularly in the consolidation or learning usually associated with NREM sleep (50).

The state dependency of vital functions is illustrated by the cessation of breathing in sleep due to neuronal deactivation in the brainstem (192, 193), pointing to the role of ascending modulatory neurotransmitter systems in the control of breathing and the generation of central apneas. Furthermore, when aggravated by age or obesity, the obstructive sleep apnea may become gravely disabling and even fatal (194). The understanding of sleep disorders may be informed by a pathophysiological approach that integrates clinical and basic biological science (195). For the mind–brain integrationist this—and other sleep disorders—provide an interdisciplinary bridge for neurologists, psychiatrists and internists. The oxygen dependence of the brain–mind is further testimony to the physical basis of waking, sleeping, and dreaming. Fortunately, sleep apnea can be treated by the application of positive airway pressure and when nocturnal brain–mind anoxia is reduced, normal waking cognition is typically restored (196).

Vital functions underwrite (and inform) belief updating from the subpersonal and interoceptive to the propositional and prosocial. This speaks to the importance of communication and psychotherapy. It is the whole brain–mind that is the target of psychotherapy. And it is sensitive to what is said. Psychological intervention is thus seen to be qualitatively akin to neurosurgery but has the twin advantages of having no structurally damaging side effects and a more global reach. This concept should delight all practitioners of talking treatment. Psychotherapy is a physical modality.

A caveat is that therapeutic efficacy must remain the gold standard of all patient treatment. It is clear that cognitive behavior therapy is the treatment of choice for most phobias—and insurance systems are wise to license it because of its efficiency and efficaciousness. But individuals of private means, who wish to investigate the psychodynamic nature of their family experience, may expect no lesser causal effects on their world view from analytically oriented psychotherapy. In fact, psychoanalytic psychotherapy may now transcend Freud's own adherence to Cartesian dualism; at the same time, the brain–mind concept fulfills his abandoned goal for a scientific psychology.

### Limitations

AIM is a preliminary model, which should be regarded as a demonstration project. Its limitations with respect to the many already well‐known alterations of conscious state have been emphasized previously. One must also appreciate the anatomical problems associated with regional brain differentiation. To be true to reality, AIM—or its derivatives—should describe brain–mind conditions at all scales, not just at the level of the whole system. Is consciousness really a winner‐take‐all phenomenon or does it consist of multiple substrates, each with its own AIM? And speaking of AIM, are three dimensions adequate to define state‐space? Almost certainly not. Skeptics ask for many more and nay‐sayers believe the brain–mind problem to be beyond the reach of science. Others may consider themselves to be descendants of Lucretius, Aristotle—or even Spinoza—and there is a clear invitation to join in this glorious conceit.
