# Correction to “Minds and Brains, Sleep and Psychiatry”

**DOI:** 10.1176/rcp2.70040

**Published:** 2026-01-30

Hobson, J.A., Gott, J.A. and Friston, K.J. (2021), Minds and Brains, Sleep and Psychiatry. *Psych Res Clin Pract*, 3: 12–28. https://doi.org/10.1176/appi.prcp.20200023.

In the article cited above, the degree of Ph.D. was attributed to Jarrod A. Gott in error.

We apologize for this error.

